# Acceptance and Commitment Therapy for Paediatric Physical Health: A Systematic Review of Child and Caregiver Outcomes

**DOI:** 10.1192/bjo.2026.11089

**Published:** 2026-06-30

**Authors:** Jamie Leveret, Louisa Wilson, Hanna Kwuo, Helen Bould

**Affiliations:** 1 University of Bristol, Bristol, United Kingdom; 2 Avon and Wiltshire Mental Health Partnership NHS Trust, Bristol, United Kingdom; 3 Royal Free London NHS Foundation Trust, London, United Kingdom; 4 Gloucestershire Health and Care NHS Foundation Trust, Gloucester, United Kingdom

## Abstract

**Aims:**

Approximately 14% of children live with a physical chronic illness (CI), which often imposes significant strain on them and the family system. Acceptance and Commitment Therapy (ACT) aims to enhance psychological flexibility, enabling individuals to remain present and pursue meaningful goals even when faced with distressing thoughts or emotions, ultimately leading to more fulfilling lives aligned with personal goals.

This review evaluated the effectiveness of ACT for paediatric patients with CI (0–18 years) and their caregivers across: parental wellbeing; parenting practices; child adjustment; and clinical markers.

**Methods:**

A systematic search of eleven databases and international trial registers was conducted for randomised controlled trials, of ACT for paediatric patients with CI (0–18 years) and/or their caregivers, published between December 2014 and July 2024. Risk of Bias (RoB) was assessed using the Cochrane RoB 2 tool via the Miguel et al. (2025) psychotherapy framework. A narrative synthesis was performed due to clinical and methodological heterogeneity. PRISMA guidelines were followed. PROSPERO CRD42021268686.

**Results:**

Eighteen reports representing 14 unique studies were identified. Overall methodological quality was judged as low, with 100% of studies rated “High Risk” or “Some Concerns” primarily driven by unblinded self-reports, high attrition (up to 60%) and widespread use of waitlist control designs.

ACT demonstrated a signal of change in reducing condition-specific affect, including caregiving-related guilt and worry in oncology (d=0.88, p=0.010) and rumination in Cerebral Palsy (p<0.01). Global distress results were inconsistent, often limited by baseline “floor effects”. Standalone ACT interventions demonstrated a positive signal of effect on parental emotional availability (specifically non-intrusiveness), parental mindfulness, comfort with diagnosis. Blinded observations of ACT noted improved child involvement (p=0.011) and reduced parental intrusiveness (p=0.050).

Gains in child quality of life and life satisfaction were observed in diabetes (p<0.001), heterogeneous CI (total HRQoL β=0.52, p=0.015), and Cerebral Palsy (social wellbeing β=10.14, p=0.025). Clinical gains included an 80% reduction in asthma-related Emergency Department visits (IRR=0.20, p=0.001) and reduced pain interference (r=−0.51, p<0.001) without corresponding shifts in absolute pain intensity (p=0.346). There was a signal for psychological flexibility as a mechanism of change present across several studies.

**Conclusion:**

ACT shows promise in shifting how families relate to chronic illness, improving quality of life and managing caregiver distress; however, overall certainty of evidence is low. Rigorous, fully powered trials utilizing blinded clinician assessments are required to confirm these exploratory signals.

